# How Complex Verbs Acquire Their Idiosyncratic Meanings

**DOI:** 10.1177/00238309231199994

**Published:** 2023-09-29

**Authors:** Sergei Monakhov

**Affiliations:** Friedrich-Schiller-Universität Jena, Germany

**Keywords:** Complex verbs, particle verbs, prefix verbs, semantic idiosyncrasy, semantic transparency, parsability, compositionality, productivity

## Abstract

Complex verbs with the same preverb/prefix/particle that is both linguistically productive and analyzable can be compositional as well as non-compositional in meaning. For example, the English *on* has compositional spatial uses (*put a hat on*) but also a non-spatial “continuative” use, where its semantic contribution is consistent with multiple verbs (*we played / worked / talked on despite the interruption*). Comparable examples can be given with German preverbs or Russian prefixes, which are the main data analyzed in the present paper. The preverbs/prefixes/particles that encode non-compositional, construction-specific senses have been extensively studied; however, it is still far from clear how their semantic idiosyncrasies arise. Even when one can identify the contribution of the base, it is counterintuitive to assign the remaining sememes to the preverb/prefix/particle part. Therefore, on one hand, there seems to be an element without meaning, and on the other, there is a word sense that apparently comes from nowhere. In this article, I suggest analyzing compositional and non-compositional complex verbs as instantiations of two different types of constructions: one with an open slot for the preverb/prefix/particle and a fixed base verb and another with a fixed preverb/prefix/particle and an open slot for the base verb. Both experimental and corpus evidence supporting this decision is provided for Russian data. I argue that each construction implies its own meaning-processing model and that the actual choice between the two can be predicted by taking into account the discrepancy in probabilities of transition from preverb/prefix/particle to base and from base to preverb/prefix/particle.

## 1 Introduction

While discussing multi-morphemic words and multi-word expressions, several important concepts should be taken into account: decomposability (analyzability, parsability), compositionality of meaning (semantic transparency), and linguistic productivity. *Decomposability* means that such linguistic expressions can be divided by language users into constituent parts and then reassembled from these parts into a whole. Cf. “[analysability is the] recognition of the contribution that each component makes to the composite conceptualisation” (Langacker, 1987, p. 292). The notion of decomposability is conditional upon the notion of *compositionality of meaning* because our ability to break a complex form into a number of simpler forms crucially depends on our ability to assign meanings to these forms (Taylor, 2012). In a similar vein, the notion of decomposability seems to imply the notion of *linguistic productivity*. As the parts of multi-morphemic words and multi-word expressions are accessible to us as form-meaning pairings, we can readily use them as building blocks to assemble new linguistic items (Laudanna, 1999).

However, if one considers a special case of multi-morphemic words / multi-word expressions—namely, complex verbs (Booij & Van Kemenade, 2003) in German and Russian, as well as verb-particle constructions in English—some problems associated with this line of reasoning become evident. In both German and Russian, there are complex-verb patterns that are (1) decomposable, (2) compositional, and (3) productive. In a prototypical situation, prefixes here encode spatial meanings, inherited from prepositions:

German(1)
*raus-gehen*
(2)
*zu-gehen*
(3)
*durch-gehen*
out-goto-gothrough-go“go out”“approach somebody”“walk through”

Russian(4)
*na-pisatj*
(5)
*nad-pisatj*
(6)
*pod-pisatj*
on-writeabove-writeunder-write“write down”“write above”“subscribe”

On the other hand, there are numerous examples of complex-verb patterns that, though totally decomposable, are not absolutely compositional in meaning (cf. Bybee, 2010, p. 45):

German(7)
*auf-klären*
(8)
*auf-bessern*
(9)
*auf-schaukeln*
on-clearon-improveon-sway“clear up”“polish up”“build up”

Russian(10)
*na-govoritj*
(11)
*na-gotovitj*
(12)
*na-rozhatj*
on-talkon-prepareon-give birth“say a lot”“cook a lot”“give birth to many”

With regard to morphological productivity, such linguistic expressions are sometimes called “semiproductive” in the literature (Jackendoff, 2002), in the sense that they (1) have some input limitations—that is, they do not accommodate every base that is semantically compatible with the preverb/prefix/particle (Blom, 2005; McIntyre, 2001)—and (2) are believed to be listed—that is, they have to be memorized. Semiproductive or not, these complex verbs often constitute very large groups of words (see the detailed discussions of specific preverb, prefix, and particle uses in German, Russian, and English in Krongauz, 1998; Kühnhold & Wellmann, 1973; Larsen, 2014; Stiebels, 1996), which, notably, are open to new members. One can compare the following examples with those listed under (7)–(9) for German and under (10)–(12) for Russian:

German(13)
*aus-merkel-n*
(14)
*rum-merkel-n*
(15)
*ver-merkel-n*
out-Merkel-INFaround-Merkel-INFpro-Merkel-INF“ignore a problem until it solves itself”“do nothing, make no decisions”“ruin, waste something completely”(all examples reflect on the political stance associated with former German chancellor Angela Merkel)

Russian(16)
*na-priviv-atj*
(17)
*na-zum-itj-sja*
(18)
*na-mitu-sh-nich-atj*
on-vaccinate-INFon-zoom-INF-REFLon-MeToo-Ø-AGT-INF“vaccinate many people”“take part in too many zoom meetings”“cause a lot of (needless) commotion while being an active part of the #MeToo movement”

Another observation about non-spatial complex verbs is that their bases are sometimes highly idiosyncratic, showing little or no semantic relation to the meaning of the complex verb (cf. English: *make off, pack off, piss off, bugger off, skive off, slope off, spirit off, bog off, push off, shove off*, and so on [examples are from McIntyre, 2002, p. 111]).

As for the meaning, some scholars argue that preverbs in verbs like German (7)–(9) and (13)–(15) or prefixes in verbs like Russian (10)–(12) and (16)–(18) are meaningless, conveying aspectual or telic interpretations (cf. Spencer & Zaretskaya, 1998). However, many complex verbs that appear to be semantically idiosyncratic when looked at in isolation reveal some interesting regularities when one studies enough verbs with the preverb or prefix in question. McIntyre (2002) argued that many non-spatial uses of particles seem to make the same semantic contribution in multiple particle verbs regardless of whether the contribution of the base is predictable (cf. *fool around* and *muck around*).

Many of these particle senses are construction-specific in that the particle’s semantic contribution is only found in verb-particle constructions and may be further limited to bases with particular semantic properties. A related idea is Zeller’s (2001) suggestion that particles are semantic affixes whose meanings are only licensed by the structural adjacency to a verb. According to McIntyre, the idiosyncrasy in particle verbs can be induced by construction-specific interpretational rules that he calls “stipulated composition rules” (McIntyre, 2002, p. 98).

Such construction-specific meanings are well attested for German, Russian, and English. In many cases, it is even possible to make hypotheses about the routes along which different preverbs/prefixes/particles developed their meanings from spatial ones onward. However, it is not perfectly clear how this construction-specific meaning is born. Even when we can identify the contribution of the base, it often seems somewhat counterintuitive to assign the remaining sememes to the preverb/prefix/particle part. For example, one could argue that, though we cannot derive the meaning of *sing on* by composing the spatial meaning of *on* with *sing*, this is not necessary for compositional analysis. We only need to assume that *on* has a second, non-spatial meaning that makes a consistent contribution to the semantics of the verb (Cappelle, 2005; Larsen, 2014). However, even if one accepts that complex verbs like *sing on* are compositional in this sense, there are other cases where one cannot speak of a “compositional” combination of a verb and particle as the root is synchronically arbitrary. For instance, *rabbit on* “talk incessantly” cannot be called compositional, as no other use of *rabbit* has a meaning associated with talking. Similar points can be made regarding combinations with *off*, such as *piss off* and others mentioned above. A Russian example might be *za-sobachitj* “hit, strike,” which is historically related to *sobaka* “dog,” but has no discernible semantic connection with this word.

These language units are parsable but not compositional in the traditional Langacker (1987) sense: the meanings of their elements can be deduced only from the general constructional meaning, and for this to be possible, the latter must be readily available.

The preverb/prefix/particle here is the main driver of the construction, and there is less focus on the base verb or element surfacing as a verb. There is, thus, more freedom to use things that are not normally verbs, and the precise semantic relation between these items and the overall meaning of the construction is less important than in a compositional scenario. This explains why many complex verbs of this type involve bases that are not normally used as verbs without the preverb/prefix/particle. Such linguistic expressions are sometimes called complex denominal verbs in the literature (Fontanals, 2001; McIntyre, 2015; Stiebels, 1998), but the thing is that they do not necessarily incorporate only nouns. Cf. deadjectival *gross someone out* “arouse disgust in someone” (< *gross* “disgusting”) or *dumb down* “simplify,” or examples where the item surfacing as a verb has no other relevant uses in the language (*divvy up* “divide something into parts or shares”). It was shown that when a certain Russian prefix-base pattern was primed in discourse, native speakers were able to arrive at the correct interpretation of even those language units in which a real prefix was combined with a nonce base (Monakhov, 2021).

The groups of complex verbs that represent established combinations of productive non-spatial preverbs/prefixes/particles with different bases can often be viewed as the clines of their nested elements’ semantic bleaching. By this, I mean that while the semantic contributions of some of these bases are easily interpretable, some other bases act more like placeholders with only expressive but no descriptive meanings (*piss off, fuck up*; cf. Brems, 2003), and still other bases no longer exist as independent words (*eke out, mete out*). It should also be noted that the arbitrariness of the bases is gradable. For example, *soldier on* (“continue to do something showing bravery, as if one were a soldier”) is more clearly motivated than *rabbit on*, and other combinations like *pipe up, key in*, or *pan out* are partly motivated for some speakers but totally arbitrary for others.

I have no diachronic evidence to support this claim, but my language intuition tells me that, at least in Russian, these clines tend to run parallel to the time axis so that the most delexicalized instances are the latest to appear. Of course, not all unmotivated bases are necessarily historically younger than fully compositional formations. There is at least one other potential source of their arbitrariness. In English, some bases originally combined with particles compositionally but then fell out of use, surviving only in particular particle verbs that were memorized. For example, *lap up* comes from the obsolete verb *lape* “drink” and *eke out* from the obsolete noun *eke* “supplement,” but today, both of them look totally idiosyncratic.

To sum up so far, apart from two clear-cut cases of linguistic expressions being either decomposable, linguistically productive, and compositional in meaning or non-decomposable, non-productive, and non-compositional, there seems to be a special third case of linguistic expressions that are decomposable, (semi?)productive and parsable: their general meanings often cannot be inferred from the meanings of their components, but the meanings of their components can be deduced from their general meanings. Complex verbs of the second, non-compositional and non-productive type are of no particular interest: they are listed diachronic relics that are retrieved from the lexicon. However, the difference between complex verbs of the first and third types merits discussion.

Monakhov (to appear) argued that there might exist two different meaning processing models for complex verbs, the distinction between which is not clear-cut and categorical but rather represents an underlying probabilistic continuum. One model implies that each of the elements entering into a combination is equally free to vary; the combination itself is judged by language users to be less semantically complex, more transparent, and tends to be more linguistically productive. Another model assigns some very general sense to the construction as such. Complex verbs of this type are very similar to collocations in the sense that they also consist of a node (conditionally independent element) and a collocate (conditionally dependent element). Such combinations of linguistic items are generally more semantically complex and less transparent because a collocate’s meaning does not coincide with the meaning of the respective free element (even if it exists) and has to be parsed out from what is available.

German and Russian provide useful insight into the problem of how the general constructional meaning of complex verbs is acquired. Derivational elements of these verbs can, generally speaking, be subsumed into two categories: spatial and non-spatial. It seems to be a general consensus that non-spatial meanings have developed from spatial ones, not only in German, Russian, and English but also in many other Indo-European languages (Amiot, 2004; Cappelle, 2005; Cuzzolin et al., 2006; Iacobini, 2009; Iacobini & Masini, 2007; Köper et al., 2016; Monakhov, 2021; Rousseau, 1995; Vincent, 1999). However, it is far from clear how exactly these processes unfolded.

I hypothesize that at the first stage of development, different preverbs/prefixes/particles with spatial meanings are combined with verbs so that they satisfy these verbs’ argument structures (McIntyre, 2007; Stiebels, 1996), thus giving rise to complex verbs whose meaning is the sum of the meanings of their parts. One can compare two very similar sentences from German and Russian (note that the provided English translation also qualifies as an example of this pattern):

German(19)
*Setzen*

*Sie*

*die*

*Zahnprothese*

*ein.*
[indenMund]putyouthedental-prothesesin[inthemouth]“Put your false teeth in. [in the mouth]”Russian(20)
*V-stavjte*

*zubnye*

*protezy.*
[vrot]in-putdentalprotheses[inmouth]“Put your false teeth in. [in the mouth]”

As one verb typically combines with many preverbs/prefixes/particles to encode different spatial meanings (as in Russian *na-pisatj* “write on,” *v-pisatj* “write in,” *nad-pisatj* “write above,” *pod-pisatj* “write under”), such instances become generalized as constructions of the form **[_____]_PREFIX_** **+** **BASE** with one empty slot and one fixed element (Diessel, 2019). Next, presumably after the number of unique bases associated with this particular preverb/prefix/particle reaches a certain threshold, a new construction of the form **PREFIX** **+** **[_____]_BASE:(X_** _**>)V**_ comes into existence by means of abstraction and categorization.^1^ This new construction then licenses certain bases to fill its empty slot, thus serving as a template with an off-the-shelf general (non-spatial) meaning for which the inserted lexical material provides a necessary specification. Importantly, some of these constructions may license the insertion of bases that have already been combined with the same preverb/prefix/particle in its spatial meaning, thus resulting in polysemous complex verbs like the German spatial *auf-nehmen* “pick up, lift up” and the non-spatial *auf-nehmen* “open, start.”

In contrast to the spatial type, non-spatial constructions do not involve the satisfaction of verbal arguments. Systematic particle uses that do not fulfill normal arguments of the verb and are sometimes labeled “adjunct-like” in the literature (McIntyre, 2007; Stiebels, 1996) are easy to find in English. Similar cases are also attested in German and Russian:

German(21)
*Man*

*kocht*

*die*

*Kartoffeln*

*vor.*
onecooksthepotatoesbefore / in front of“One prepares potatoes ahead of time.”Russian(22)
*On*

*za-gotovil*

*mnogo*

*edy*

*vprok.*
hebefore/behind-prepareda lot offoodfor the future“He prepared a lot of food for the future.”

I interpret the process whereby complex verbs of this type are produced as an instantiation on the morphological level of the so-called “semantic coherence principle” of construction grammar (Goldberg, 2006) which implies that constructions attract lexical items compatible with the semantic specifications of certain slots. In particular, this means that each specific complex verb with idiosyncratic meaning must be construable as an instance of the more general construction of the form **PREFIX** **+** **[_____]_BASE:(X_** _**>)V**_. Following this line of reasoning, one can account for the fact that non-spatial complex verbs with a certain preverb/prefix/particle often come in groups of numerous members such that the meanings of derivations are almost identical, although the meanings of their bases might have nothing in common. I believe this analysis is non-contradictory to both Zeller’s idea of particles as semantic affixes that have certain selection restrictions stipulating the kinds of verbs they can combine with (Zeller, 2001) and Stiebels’s (1996) theory of lexical operations that can change a verb’s lexical entry to allow it to accommodate a particle.

The rest of this paper is dedicated to testing, on Russian data, the hypothesis that complex verbs with spatial and non-spatial meanings represent two different constructions. The paper is structured as follows. In study 1, I provide experimental evidence that native speakers, when asked to manipulate complex verbs by changing either their prefix or their base, reveal significant preferences for changing the prefixes of spatial verbs and the bases of non-spatial verbs.

In study 2, I draw on the idea that one can identify the construction of a specific complex verb by estimating the ratio of two transitional probabilities: P (prefix | base) and P (base | prefix). I provide empirical, corpus-based evidence that for spatial complex verbs, the probability ratio P (prefix | base) / P (base | prefix) is, on average, less than 1, while for non-spatial verbs, it is greater than 1. I interpret this difference as confirming the hypothesis of two constructions—**[_____]_PREFIX_** **+** **BASE** and **PREFIX** **+** **[_____]_BASE:(X_** _**>)V**_—as, intuitively, one would expect to find that the fixed element communicates less information about the filler than the filler communicates about the fixed element (cf. Gries & Stefanowitsch, 2004).

In the first part of study 2, I show that the transitional probabilities ratios obtained for 2,566 Russian complex verbs are strongly negatively correlated with these verbs’ compositionality scores, which is in line with my initial assumption that complex verbs of the form **PREFIX** **+** **[_____]_BASE:(X_** _**>)V**_ cannot be called compositional in the traditional sense. In the second part of study 2, two linear regression models are trained to predict the degree of compositionality of a given verb using either the ratio of transitional probabilities or the derivation to base frequency ratio, an alternative measure proposed by Hay (2001, 2003) for assessing the degree of decomposability of multi-morphemic words. The greater predictive power of the former is reported.

## 2 Study 1. Experimental evidence of the existence of two complex verbs’ constructions

### 2.1 Hypothesis

One simple way to see whether spatial and non-spatial prefixed verbs are processed differently is to provide native speakers with a respective linguistic item and ask them to write down the first word they can think of that differs from the presented word by either its prefix or its base. If verbs with spatial meanings are indeed constructions of the form **[_____]_PREFIX_** **+** **BASE** with an empty slot for the prefix, then such a test will reveal participants’ preference to manipulate derivational elements of these expressions to produce words with the same base but with different prefixes. Conversely, if verbs with non-spatial meaning are, as I hypothesize, constructions of the form **PREFIX** **+** **[_____]_BASE:(X_** _**>)V**_ with an empty slot for the base, then participants will most likely keep the prefix, as a fixed element, unchanged and manipulate the base.

### 2.2 Stimuli

Shvedova (1980) lists 28 verbal prefixes in Russian, of which:

- 17 prefixes are not only historically related to prepositions but also have prepositional counterparts in modern Russian: *v-* (*v* “in, at”), *do-* (*do* “to, before”), *za-* (*za* “for, behind”), *iz-* (*iz* “from, out of”), *na-* (*na* “on”), *nad-* (*nad* “over, above”), *o-* (*o* “about”), *ob-* (*ob* “about”), *ot-* (*ot* “from”), *po-* (*po* “along, by”), *pod-* (*pod* “under”), *pred-* (*pered*/*pred* “before, in front of”), *pri-* (*pri* “by, at”), *pro-* (*pro* “about, of”), *s-* (*s* “with”), *so-* (*so* “with”), and *u-* (*u* “from, by”);
- 11 prefixes have no prepositional counterparts in modern Russian; this group encompasses morphemic borrowings, prefixes that have non-prepositional origin and prefixes derived from prepositions that are no longer part of the Russian language: *de-, dis-, vz-, voz-, vy-, nedo-, niz-, pere-, pre-, raz-*, and *re-*.

Almost all Russian verbal prefixes, both prepositional and non-prepositional, are polysemous, with the number of meanings ranging from 2 (e.g., *v*-) to 10 (e.g., *pere*-). For the experiment, all meanings of all prefixes listed by Shvedova (1980) were taken into consideration: 91 meanings for prepositional prefixes and 34 meanings for non-prepositional prefixes—125 in total. For each meaning, one verb was randomly selected from the list of examples provided by Shvedova (1980). The whole set of experimental stimuli can be found in Appendix.

### 2.3 Experimental design and participants

The experiment was completed online. I used Yandex Toloka, a Russian crowdsourcing service analogous to Amazon Mechanical Turk, to conduct the experiment. Instructions for the participants read as follows (translated from Russian):

In each task, you will be given one Russian prefixed verb. Please write, in each case, the first verb you can think of that differs from the presented one by either its prefix or its base. Please note that your input must contain either the same prefix and a different base or the same base and a different prefix. Otherwise, the assignment will not be accepted.

Each verb was presented in 30 tasks to 30 different people. Yandex Toloka does not grant access to their workers’ personal data but allows for some coarse-grained social stratification while assembling pools of users. I made sure that the number of males and females in the set of participants was approximately equal, their age ranging from 18 to 55, all of them having obtained at least upper secondary education. The overall number of tasks equaled 3,750 (125 verbs x 30). A total of 186 native speakers of Russian took part in the experiment, and each participant worked with a random selection of 19–22 verbs.

### 2.4 Analysis of the results

Ultimately, 166 submissions were excluded as non-conforming to the instruction, resulting in 3,584 accepted answers. For example, for the verb *ot-gremetj* “stop rumbling,” the following entries were submitted:

- 22 instances of verbs with a different prefix and the same base, among them: *po-gremetj* “rumble for a while” (two instances), *pro-gremetj* “emit a rumbling sound” (six instances), *za-gremetj* “start rumbling” (14 instances);
- seven instances of verbs with a different base and the same prefix, among them: *ot-pravljatj* “send away,” *ot-kalyvatj* “chip away,” *ot-gruzhatj* “load, ship,” *ot-zvenetj* “stop ringing,” *ot-vertetj* “screw off,” *ot-davatj* “give back,” *ot-letatj* “stop flying.”

I am now interested in how much variability, or uncertainty, there is in the prefix and base part of the results. One simple way to determine this is to calculate, separately, the prefix and base entropy by applying the formula

$$E\left(X\right)=-\sum_{i=1}^{N}p\left(x_{i}\right)log_{2}\left(p\left(x_{i}\right)\right).$$

The concept of entropy is used to refer to the measure of randomness or disorder within a system. The formula above clearly shows why entropy is also called the measure of “expected surprise.” Consider two opposite cases. First, there may be a system where many states are equiprobable, which means that their probabilities (relative frequencies) are comparably low. In this case, one’s surprise at finding the system in a particular state will always be great because the process of change is random, and no expectations are formed. Alternatively, there might be a system in which one state is much more probable than the others. In this case, one would expect to find the system in its favorite state and would not be surprised if this expectation was confirmed. Obviously, negative logarithms of small probability values are greater than negative logarithms of high probability values, so the measure of expected surprise (entropy) will be greater for highly disordered systems than for stable systems.

In the previous example with the stimulus *ot-gremetj*, there are four unique prefixes in the output that are used the following number of times each (Table 1):

**Table 1.** Calculating the Prefix Entropy of Experimental Results for the Verb *ot-gremetj*.

Prefixes	*za-*	*ot-*	*po-*	*pro-*
Counts	14	7	2	6
Probabilities	.48	.24	.07	.21

Applying the formula given above, one can calculate the measure of entropy: 1.20. This logic readily extends to the bases. There are eight unique bases in my example that are used the following number of times each (Table 2):

**Table 2.** Calculating the Base Entropy of Experimental Results for the Verb *ot-gremetj*.

Bases	*vertetj*	*gremetj*	*gruzhatj*	*davatj*	*zvenetj*	*kalyvatj*	*letatj*	*pravljatj*
Counts	1	22	1	1	1	1	1	1
Probabilities	.034	.76	.034	.034	.034	.034	.034	.034

As can be seen from the numbers, one base—namely, *gremetj*—totally dominates the distribution, and so the entropy value for the base part of the example is predictably lower than the prefix part’s entropy: 1.02.

**Figure 1.**
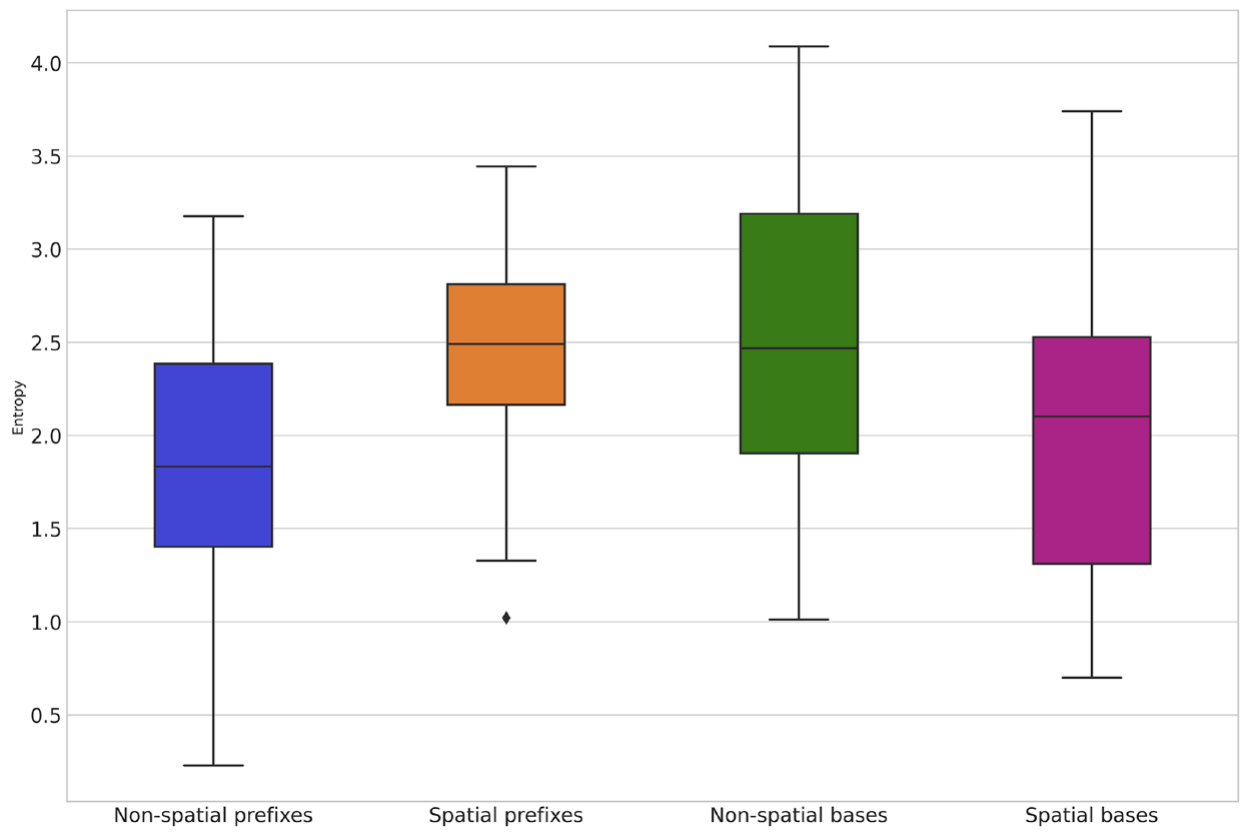
Boxplots of the entropy values.

Now, let us return to the initial hypothesis. What one would expect given the aforementioned idea of the two constructions can be stated as follows: for the verbs with prefixes encoding spatial relations, prefix entropy will be higher than for the verbs with prefixes encoding non-spatial, derived relations, as the former have an empty slot for the prefix. Conversely, base entropy will be higher for the verbs with prefixes encoding non-spatial relations than for the verbs with prefixes encoding spatial relations, as the former have an empty slot for the base. The distributions of the prefix and base entropy values for all the words in my dataset are visualized in Figure 1.

The statistics and *p* values of the *t*-tests for independent samples are presented in Table 3. They are shown separately for the words with prepositional prefixes, the words with non-prepositional prefixes, and all prefixes without regard to their etymology. In the last row of the table, one can find the median ratios of each word’s prefix entropy value to its base entropy value.

**Table 3.** Inferences on the Difference of Mean Entropy Values Between Verbs With Spatial and Non-Spatial Meanings.

	All prefixes		Prepositional		Non-prepositional	
	Spatial	Non-spatial	Spatial	Non-spatial	Spatial	Non-spatial
Prefix entropy	*t* = 4.51 (*p* < .01)		*t* = 3.07 (*p* < .01)		*t* = 3.41 *(p* < .01)	
Base entropy	*t* = −3.60 (*p* < .01)		*t* = −2.72 (*p* < .01)		*t* = −2.07 (*p* = .04)	
Median ratio	1.26	0.72	1.29	0.77	1.11	0.65

It is clear that the null hypothesis that there would be no difference between the prefix and base entropy values of Russian verbs with spatial and non-spatial meanings can be safely rejected. Significant differences were observed not only for the verbs whose prefixes coincide in form with existing prepositions but also for the verbs whose prefixes do not exist as free morphemes. This means that the empty slot in the construction **[_____]_PREFIX_** **+** **BASE** can be filled both with elements corresponding to real prepositions and with elements that are parsed out from other complex verbs.

I expect that similar results would be observed both (1) in German, where compositional stimuli like *raus-gehen* “go out” will produce suggestions like *zu-gehen* “approach somebody” or *durch-gehen* “walk through” and idiosyncratic stimuli like *an-braten* “sear, brown” will produce suggestions like *an-brennen* “light, burn,” *an-knabbern* “nibble at,” or *an-kratzen* “scratch, dent”; and (2) in English, where stimuli like *put down* will result in suggestions like *put on* or *put under* and stimuli like *brush down* will result in suggestions like *clean down, scour down*, or *scrub down*.

## 3 Study 2. Corpus evidence in favor of the two-construction account

### 3.1 Degrees of compositionality and transitional probabilities

The aim of this study is to show that the difference between two complex verbs’ constructions under investigation manifests itself in the difference between the prefix → base and base → prefix transitional probabilities of the respective complex verbs. I provide evidence that the ratios of these transitional probabilities correlate with the complex verbs’ compositionality scores obtained from corpus data and from a word-embedding model in a way supporting my initial assumption that spatial verbs instantiate constructions of the form **[_____]_PREFIX_** **+** **BASE** and idiosyncratic verbs instantiate constructions of the form **PREFIX** **+** **[_____]_BASE:(X_** _**>)V**_.

In computational linguistics, one of the long-standing problems of sense disambiguation is the automatic prediction of literal versus non-literal language usage (Gutierrez et al., 2016; Hamilton et al., 2016; Schlechtweg et al., 2017; Turney et al., 2011; Wang et al., 2018). With regard to prefixed verbs, this distinction is epitomized by the division of verbs into groups encoding spatial meanings and verbs encoding non-spatial meanings. I hypothesize that prefixed verbs encoding spatial meanings represent constructions that have an empty slot for prefixes and tend to be compositional, which means that both elements in combination contribute to a general, additive meaning. However, non-spatial, idiosyncratic verbs represent constructions that have an empty slot for bases. They tend to be non-compositional but parsable: some very general sense is assigned to the construction as such, which results in the meaning of a filler base not coinciding with the meaning of a respective free element (even if it exists) and needing to be parsed out from what is available.

The very nature of these two constructions, with their reversed positioning of the fixed element and the empty slot, suggests that one can identify the construction to which each particular word belongs by estimating two probabilities: P (prefix | base) and P (base | prefix). Suppose that P (prefix | base) / P (base | prefix) = *ε*. Then, a linguistic item is more likely to be of the form **[_____]_PREFIX_** **+** **BASE** if *ε* ⩽ 1 and more likely to be of the form **PREFIX** **+** **[_____]_BASE:(X_** _**>)V**_ if *ε* > 1. I expect it to be this way and not the other way around because I assume that the fixed element communicates less information about the filler than the filler communicates about the fixed element. Thus, P (prefix | base) must be greater than P (base | prefix) with idiosyncratic verbs and smaller than P (base | prefix) with spatial verbs.

Based on these premises, one might hypothesize that it would be possible to predict the degree of compositionality of a given verb by taking into account its ratio of transitional probabilities, P (prefix | base) / P (base | prefix).

The probabilities P (prefix | base) and P (base | prefix) can be evaluated empirically, for example, by taking all of the prefixed verbs in a morphemic dictionary of the respective language and looking up the frequencies of interest in the internet corpus of this language. Then, for any word, its P (prefix | base) is equal to the number of that word’s tokens divided by the number of tokens of all (prefixed) words with this base, and P (base | prefix) is equal to the number of the given word’s tokens divided by the number of tokens of all words with this prefix.

To build a model capable of predicting complex verbs’ compositionality degrees based on their transitional probabilities P (prefix | base) and P (base | prefix), I obtained all Russian prefixed verbs included in the *Word-Formation Dictionary of the Russian Language* (Tikhonov, 1985). Overall, there were 6,159 verbs. I decided to constrain the task to verbs with prepositional prefixes for the following reasons. To train the model, I need some objective measure of how much spatial meaning a certain prefixed verb encodes. Given the amount of data, manual coding seemed infeasible. Therefore, one option was to get an approximation of this measure from a linguistic corpus, relying on the fact that verbs with spatial meanings encoded by prepositional prefixes are often accompanied by prepositions in Russian, unlike their counterparts with idiosyncratic meanings (cf. Bergsma et al., 2010; Biskup, 2015, 2019):

(23)
*na-pisatj*

*na*

*bumage*
on-writeonpaper“write on paper”(24)
*na-vratj*

*o*

*proizoshedshem*
on-lieaboutwhat-happened“lie a lot about what has happened”

Hence, only verbs with prepositional prefixes could be included in the survey. In my data, there were 4,580 such verbs. Using *ruTenTen11*, an internet corpus of Russian from 2011 provided by Sketch Engine and containing more than 14 billion words (Jakubíček et al., 2013), I queried two types of co-occurrence frequencies for each verb: (1) one where a preposition coinciding in form with the verbal prefix is found within the window of four words to the left of the verb, and (2) another where the same preposition is found within the window of four words to the right of the verb. The choice of window size is consistent with the rules of thumb frequently suggested in the literature. These are based on the observation that a smaller window size focuses on how the word is used and learns what other words are functionally similar to it, whereas a larger window size captures information about the domain or topic of each word (Hvitfeldt & Silge, 2022; Levy & Goldberg, 2014; Lin et al., 2015). All prefixal and prepositional allomorphs were queried in the corpus separately; the numbers were then combined.

To calculate the final measure of compositionality for each verb, one must control for the verb’s and the preposition’s overall frequency of use because highly frequent verbs and highly frequent prepositions may often co-occur by random chance. To do this, I used the logDice score, a metric from corpus linguistics that is designed to measure collocation strength (Rychlý, 2008). The logDice score has the following useful features: (1) the score does not depend on the total size of a corpus; (2) its theoretical maximum is 14, in cases when all instances of word A co-occur with word B and all instances of word B co-occur with word A; and (3) its negative values mean that there is no statistical significance of the collocation of words A and B.

This metric is easy to calculate and interpret. For example, the total number of co-occurrences of the verb *na-brositj* “throw on(to)” and the preposition *na* “on” is 3,206. The preposition *na* has an overall frequency of 2.41e8, and the verb *na-brositj* has an overall frequency of 8,388. The logDice score of this combination of words is 3.465. This means that the verb *na-brositj* and the preposition *na* tend to appear alongside each other. Hence, one can conclude that *na-brositj* is likely to encode compositional spatial meaning. However, the verb *na-petj* “sing along” is randomly seen in the vicinity of the preposition *na* (logDice score equal to -0.263), which supports the native speaker’s intuition about the general constructional meaning of this linguistic item being non-spatial.

Table 4 provides some further results for illustration. From this table, it becomes clear that, evaluated as I suggest, the compositionality of Russian prefixed verbs can be viewed as a continuum, with some of the verbs retaining much of the prepositional spatial meaning and some drifting far away from it.

**Table 4.** logDice Scores for Some Russian Verbs with the Prefix *na-*.

Verb	Meaning	logDice score
*na-pisatj*	“write on”	7.324
*na-brositj*	“throw on(to)”	3.465
*na-valitj*	“pile up”	1.844
*na-lovitj*	“catch a lot”	0.382
*na-petj*	“sing along”	−0.263
*na-soritj*	“litter”	−1.349

**Figure 2.**
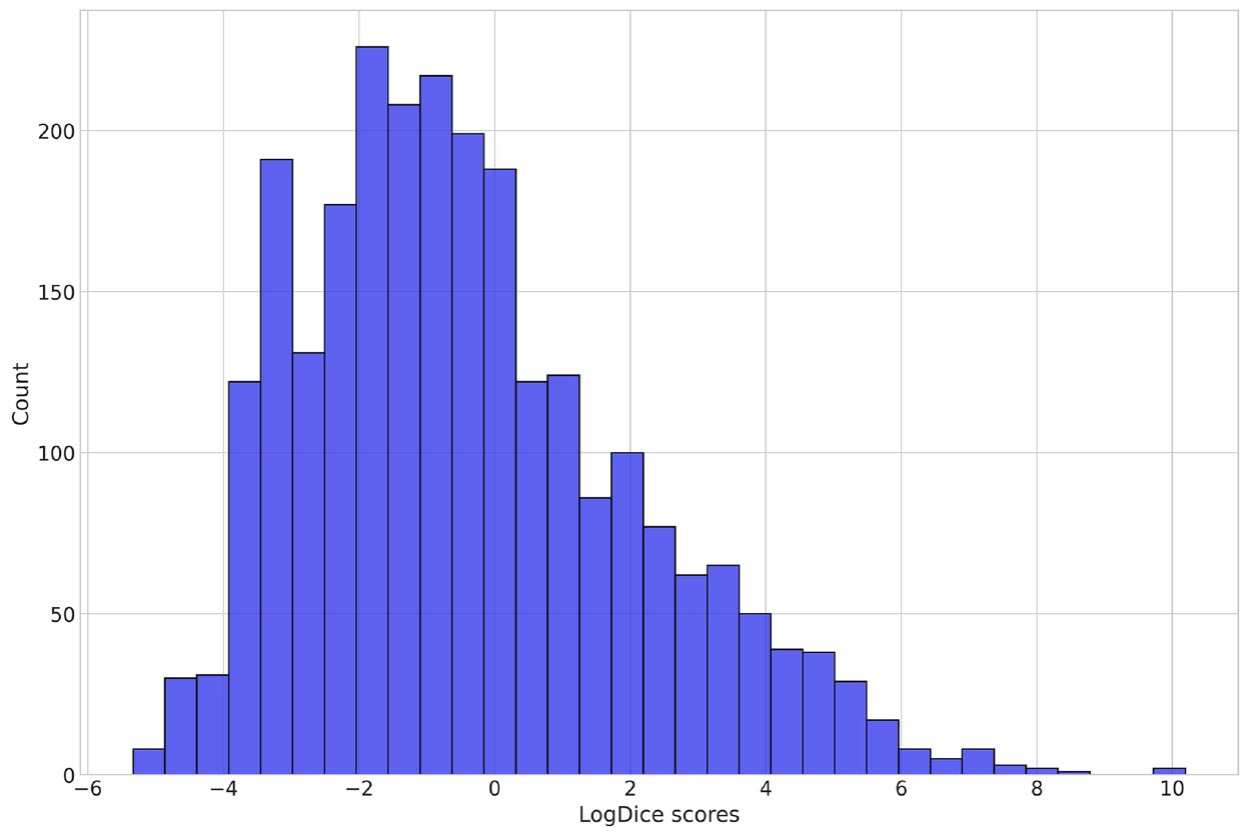
Histogram of the logDice scores.

One problem with the logDice score is that it is undefined for the case of no co-occurrence of a particular combination of words because this requires taking a log of 0. Therefore, I had to exclude all the verbs for which not a single instance of the preposition that coincides in form with the verbal prefix was found within the specified window in the whole corpus. This is justified by the fact that, given the ubiquitousness of prepositions, for sufficiently frequent verbs, at least some co-occurrences should happen by random chance. After pruning, there were 2,566 complex verbs left in the dataset. The distribution of the verbs’ compositionality measures (logDice scores) can be found in Figure 2.

Before creating a predictive model, I wanted to make sure that there indeed exists some relationship between Russian complex verbs’ degrees of compositionality and transitional probabilities ratios. Once again, my hypothesis implied that for the prefixes that encode mostly spatial meanings, there would be many verbs with P (prefix | base) ⩽ P (base | prefix) so that the ratio of the two, averaged across all items, would be less than 1 (or 0 on the log scale). Alternatively, with the prefixes that encode mostly non-spatial meanings, one would find many lexemes with P (prefix | base) > P (base | prefix) so that the ratio of the two, averaged across all items, would be greater than 1.

To illustrate, let us consider two Russian verbs with the same prefix *v-: v-chinitj* “submit, file (a complaint)” and *v-bezhatj* “run into.” The values of interest for each of them are given in Table 5.

**Table 5.** Exemplary Calculations for the Verbs *v-chinitj* and *v-bezhatj*.

Values	*v-chinitj*	*v-bezhatj*
L—number of word’s tokens	485	17,455
S—number of tokens of all words with this base	512,401	1,768,006
P—number of tokens of all words with this prefix	753,546	753,546
P (prefix | base) = L / S	0.0009	0.009
P (base | prefix) = L / P	0.0006	0.02
ratio P (prefix | base) / P (base | prefix)	1.47	0.42
log ratio P (prefix | base) / P (base | prefix)	0.38	−0.85
logDice score	−3.72	3.48

Based on these calculations, I would expect to find that if the prefix-base construction with *v-* encodes mostly spatial meanings, there will be many verbs like *v-bezhatj*; otherwise, there will be many verbs like *v-chinitj*. The general picture for all prefixes in my data will then be that of the negative correlation between average compositionality measures and average transitional probabilities ratios (log-transformed).

**Figure 3.**
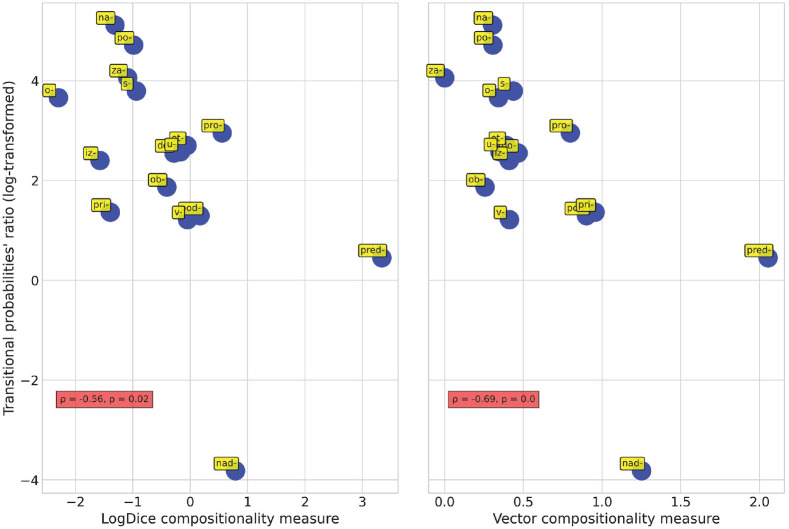
Correlation of transitional probabilities ratios with compositionality measures.

Such a correlation was indeed observed (Figure 3, left-hand panel; ρ = −0.56, *p* = .02). We can easily convince ourselves that prefixes with a high degree of compositionality (*pred-, nad-, pod-, v-*) are characterized by low values of transitional probabilities ratios, which signifies that for the verbs with these prefixes, on average, P (prefix | base) ⩽ P (base | prefix). However, prefixes that represent constructions that have acquired numerous non-spatial meanings over the course of their development (*na-, o-, po-, s-/so-, za-*) reveal high values of transitional probabilities ratios, which signifies that for the verbs with these prefixes, on average, P (prefix | base) > P (base | prefix).

Up to this point, I had estimated transitional probability P (prefix | base) as a number of a certain word’s tokens divided by the number of tokens of all (prefixed) words with the respective base and transitional probability P (base | prefix) as a number of a certain word’s tokens divided by the number of tokens of all words with the respective prefix. One might wonder whether the relationship between transitional probabilities ratios and compositionality measures is dependent upon the frequency of complex verbs and their bases. This can be easily determined by estimating the same two transitional probabilities from type rather than token frequencies.

P (prefix | base) can be estimated as 1 divided by the number of prefixes that combine with a given base, and P (base | prefix) as 1 divided by the number of bases that combine with a given prefix. By performing these calculations on the dataset of Russian prefixed verbs, I ascertained that type- and token-based transitional probabilities ratios are almost perfectly correlated (ρ = 0.9, *p* < .001), which means that P (TP_*i*_ | C_*i*_, F_*i*_) = P (TP_*i*_ | C_*i*_), where *C_i_* is the compositionality measure of a prefixed verb *i*, TP_*i*_ is this verb’s transitional probabilities ratio, and F_*i*_ is its token frequency.

### 3.2 Distributional semantic estimates of the degree of compositionality

My assumption that Russian prefixed verbs collocating with prepositions necessarily encode spatial meanings might sound too strong. Indeed, in some cases, unexpectedly high compositionality scores were attested for the verbs with obviously non-spatial meaning. For example, the verb *za-rezatj* “slaughter, kill with a knife” has a relatively high logDice score of 2.01, and the score of another verb with the same base, *na-rezatj* “slice,” is even higher, at 5.44. However, there is nothing compositional in these verbs’ senses. High logDice scores for *za-rezatj* and *na-rezatj* are due to the fact that these words frequently co-occur with prepositions that do not encode spatial meanings themselves, as in the following examples:

(25)
*na-rezatj*

*na*

*kuski*
on-cutintopieces“slice into pieces”(26)
*za-rezatj*

*za*

*kopejku*
before/behind-cutforkopeck“slaughter for nothing”

The number of such cases is relatively small and is unlikely to undermine the validity of my conclusions in general. However, I wanted to find out whether the results would hold with a different compositionality measure.

An alternative way of obtaining complex verbs’ compositionality measures is suggested by the distributional hypothesis, which states that similarity in meaning results in similarity in linguistic distribution (Firth, 1957). Words that are semantically related tend to be used in similar contexts. Hence, by reverse-engineering this process—that is, coding words’ discourse co-occurrence patterns with multi-dimensional vectors and performing certain algebraic operations on them—distributional semantics can induce semantic representations from contexts of use (Boleda, 2020). It is well-established that the similarity of words’ vector representations goes beyond simple syntactic regularities (Mikolov et al., 2013; Pennington et al., 2014; Rehurek & Sojka, 2011) and that vector space models perform well on tasks that involve measuring the similarity of meaning between words, phrases, and documents (Turney & Pantel, 2010).

More importantly, for the purposes of my study, vector space models have been used for assessing the degree of compositionality of complex linguistic expressions, notably nominal compounds (Cordeiro et al., 2019) and particle verbs in English (Bannard, 2005) and German (Bott & Schulte im Walde, 2014). These analyses generally assume that multiword expressions are highly variable in compositionality and that if the meanings of some of them can be described as the sum of the meanings of their parts, then a distributional semantic model will reveal significant similarity between vectors for a compositional expression and for the combination of the vectors of its parts, computed using some vector operation. Conversely, the lack of such similarity might be interpreted as a manifestation of complex expressions’ idiomaticity.

Applying the aforementioned principle to multi-morphemic words, specifically to Russian complex verbs whose prefixes have corresponding free elements, seems a straightforward extension. My hypothesis is that one can model the difference between spatial and non-spatial prefixed verbs by performing simple algebraic operations on semantic vectors representing the target verbs and their subparts. Specifically, I am interested in estimating the ratio **cosine** $(\vec{V},$ $\vec{C})$ / **cosine** $(\vec{V},$ $\vec{P}),$ where $\vec{V}$ is a vector for the verb, $\vec{C}$ is a “compositionality operation vector” obtained by summation of the vectors for the base and preposition coinciding in form with the prefix, and $\vec{P}$ is a “parsability operation vector” obtained by subtraction of the vector for the base from the verb vector $\vec{V}.$ I hypothesize that this ratio will be greater for the prefixed verbs that are instantiations of the **[_____]_PREFIX_** **+** **BASE** constructions and smaller for the prefixed verbs that are instantiations of the **PREFIX** **+** **[_____]_BASE:(X_** _**>)V**_ constructions.

While the assumption about the results of the vector addition operations is intuitively clear, it might not be obvious why I believe that the results of the vector subtraction operations will be of any importance. As discussed in the introduction, my hypothesis implies that constructions with an empty slot for the prefix are instantiated by spatial complex verbs with compositional meaning, and hence, the meaning of the whole expression, in this case, can be most adequately represented as the sum of the meanings of the prefix and the base (“compositionality operation vector”). In contrast, constructions with an empty slot for the base are instantiated by complex verbs with idiosyncratic meaning, such that the prefix is the main driver of the construction, and the base only provides the necessary specification for the general constructional meaning. Hence, even if we remove this semantic specification component (“parsability operation vector”), this procedure should not be completely detrimental to the composite conceptualization.

To demonstrate, let us consider two groups of 10 nearest neighbors (i.e., words whose vector representations and, by extension, co-occurrence patterns are most similar to the target word) of the vectors obtained as a result of (1) subtraction of the vector of the base *pisatj* “write” from the vector of the verb *na-pisatj* “write on” and (2) subtraction of the vector of the base *lovitj* “catch” from the vector of the verb *na-lovitj* “catch a lot” (Table 6). The vectors for this example were obtained from the word2vec continuous skip-gram model provided by the *RusVectōrēs* project (https://rusvectores.org; Kutuzov & Kuzmenko, 2017). The model includes functional words and was trained on the Russian National Corpus and the Russian Wikipedia dump of 2018.

**Table 6.** Output of the Vector Subtraction Operations on the Verbs *na-pisatj* and *na-lovitj*.

*na-pisatj*		*na-lovitj*	
Nearest neighbors of $\vec{P}$	Cosine similarity $(\vec{V},$ $\vec{P})$	Nearest neighbors of $\vec{P}$	Cosine similarity $(\vec{V},$ $\vec{P})$
*na-pisatj* “write on”	.48	*na-lovitj* “catch a lot”	.69
*kaver-versii* “cover.PL”	.33	*na-rubitj* “chop a lot”	.40
*pozhalujsta* “please”	.32	*na-streljatj* “shoot a lot”	.38
*spoj* “sing.IMP”	.32	*na-pech* “bake a lot”	.37
*davajte-ka* “let us”	.31	*na-kopatj* “dig a lot”	.33
*Neil Young*	.31	*na-gotovitj* “cook a lot”	.33
*vos-hoditj* “rise”	.31	*s-varitj* “boil”	.33
*za-pisatj* “record”	.31	*s-vezti* “bring together”	.33
*Cliff Richard*	.30	*po-zharitj* “fry”	.33
*nu-ka* “come-on.INT”	.30	*zakusochka* “little snack”	.32

*Note.* Cosine similarity ranges from 0 to 1 for vectors with positive values.

Intuitively, the numbers in Table 6 tell us that prefixed verbs with spatial meanings, like *na-pisatj*, strongly overlap in semantics and distribution with their bases. For this reason, there is almost nothing meaningful left in the vectors of these verbs after the key components have been subtracted. The output in the left-hand panel of Table 6 contains mostly irrelevant noise, including proper nouns like *Neil Young*, interjections like *nu-ka* “come-on.INT,” imperative or hortative forms like *davajte-ka* “let us,” and so on.

The situation is very different with non-spatial prefixed verbs like *na-lovitj*. Here, as the items in the right-hand panel of Table 6 suggest, the result of vector subtraction does encode some conceptual entity of its own—some very general sense that is attributed to the construction as such (consider the variability of specific lexical meanings in the set of the words aligned with *na-lovitj: na-rubitj, na-streljatj, na-pech, na-kopatj, na-gotovitj*).

Note that the vectors obtained by subtraction also show significant differences in terms of their cosine similarities to the initial lemmas’ vectors: 0.48 for *na-pisatj* and 0.69 for *na-lovitj*. The cosine similarities of the initial lemmas’ vectors and the vectors obtained by summation (preposition + base) are also different, but this difference goes in the opposite direction: 0.57 for *na-pisatj* and 0.48 for *na-lovitj*. Thus, the ratio **cosine** $(\vec{V},$ $\vec{C})$ / **cosine** $(\vec{V},$ $\vec{P})$ is equal to 1.18 for *na-pisatj* and to 0.69 for *na-lovitj*, exactly as I expected.

Some words of caution may be appropriate at this point. Vector space models have well-known limitations. Specifically, traditional word2vec (Mikolov et al., 2013) and GloVe (Pennington et al., 2014) models tend to perform worse when confronted with word formation in morphologically rich languages like German (Köper et al., 2015) and Russian (Drozd et al., 2016). Thus, of all the existing non-contextualized pretrained vector models of the Russian language, the FastText model seemed the best suited for the purposes of this study. While other popular models ignore the morphology of words by learning their vectors, in the FastText model, a vector representation is associated with each character *n*-gram, and words are represented as the sums of these *n*-gram vectors (Bojanowski et al., 2017).^2^

Using this model, I obtained for each verb in my data its **cosine** $(\vec{V},$ $\vec{C})$ / **cosine** $(\vec{V},$ $\vec{P})$ ratio and then averaged the values prefix-wise. The results of the correlation analysis of these measures with the log-transformed transitional probabilities ratios are visualized in Figure 3 (right-hand panel). The strength of association is even greater than the one observed for the logDice scores, but the direction of association is the same, in line with my expectations (ρ = −0.69, *p* = .002).

One might argue that distributional semantic models represent abstractions over attested data, and further modifying the vectors for assessing multi-morphemic words’ degrees of compositionality is yet another step away from the empirical basis. However, while each of my two measures of compositionality on its own may be considered problematic in some respects, the fact that they, having been obtained independently, agree with each other so well should greatly reinforce one’s confidence in either of them. In fact, the similarity between the two plots in Figure 3 is truly remarkable and suggests that both proposed ways of measuring Russian prefixed verbs’ degrees of compositionality are reliable.

### 3.3 Automatic prediction of Russian prefixed verbs’ spatial and non-spatial meanings

This section will return to the idea of building a predictive model of the prefixed verbs’ degrees of compositionality. Taking into account the observed negative correlation between the compositionality measures and transitional probabilities’ ratios of Russian prefixed verbs, I hypothesize that individual words with greater compositionality scores (those more likely to encode spatial meanings) would be characterized by a P (prefix | base) approximately equal to or lower than their P (base | prefix), whereas words that have lower compositionality scores (those more likely to encode non-spatial, construction-specific meanings) would tend to reveal P (prefix | base) values that are greater than their P (base | prefix) values.

It would also be interesting to compare the accuracy of this model with the accuracy of another model built for the same purposes but basing its decisions not on transitional probabilities ratios but on the derivation to base frequency ratio, the measure proposed by Hay (2001, 2003). The logic behind this measure is as follows. According to Hay, the degree of decomposability of a given item depends on the frequency of the derived word relative to its base. With most complex words, the base is more frequent than the derived form, so the relative frequency is less than 1. Such words, Hay argues, are more easily decomposed; that is, they are more likely to be accessed via a morpheme-based route. In the opposite case, when the derived form is more frequent than the base, a whole-word bias in parsing is expected. This has consequences for semantics, and such words become less transparent and more polysemous.

Given Hay’s method of measuring linguistic relativity, one might hypothesize that it would be possible to automatically classify prefixed verbs encoding spatial and non-spatial meanings by assigning decomposable lexemes with Frequency(base) > Frequency(prefix + base) to the first group and non-transparent lexemes with Frequency(base) < Frequency(prefix + base) to the second group. Comparing the accuracies of these two models will show which way of estimating complex verbs’ degree of analyzability is more accurate—the one based on transitional probabilities ratio or the one based on derivation to base frequency ratio.

To illustrate, let us consider two Russian verbs with the same base *rezatj* “cut”: *ot-rezatj* “cut off from” and *za-rezatj* “slaughter, kill with a knife.” The values of interest for each of them are given in Table 7.

**Table 7.** Exemplary Calculations for the Verbs *ot-rezatj* and *za-rezatj*.

Values	*ot-rezatj*	*za-rezatj*
P (prefix | base)	0.095	0.019
P (base | prefix)	0.117	0.009
ratio	0.095 / 0.117 = 0.806	0.019 / 0.009 = 2.088
logDice score	7.03	2.01
Hay’s parsability measure	201,598 / 199,753 = 1.009	38,002 / 199,753 = 0.19

One can say that these two words epitomize the distinction between spatial and non-spatial types. The verb *ot-rezatj* is an instantiation of the construction of the form **[_____]_PREFIX_** **+** **BASE**. Its meaning can be construed as the sum of the meanings of the base and respective preposition *ot* “away, from.” We can easily convince ourselves that this meaning is compositional by looking at the verb’s very high logDice score of 7.03. The verb *za-rezatj*, however, is different in that it instantiates the **PREFIX** **+** **[_____]_BASE:(X_** _**>)V**_ construction, which suggests “to bring someone to an undesirable state (of unfitness, fatigue, exhaustion, death) through an action identified by the base.” This construction’s general meaning is obviously non-compositional as it does not inherit anything from the meaning of the corresponding preposition *za* “behind,” which is confirmed by a significantly lower logDice score of 2.01.

The ratios of transitional probabilities seem to capture this semantic difference correctly. The ratio of *ot-rezatj*, 0.806, is much smaller than the ratio of *za-rezatj*, 2.088. This is in line with my hypothesis that for the prefixes that encode mostly spatial meanings, there will be many verbs with P (prefix | base) ⩽ P (base | prefix), whereas with the prefixes that encode mostly non-spatial meanings, one will find many lexemes with P (prefix | base) > P (base | prefix).

Surprisingly, Hay’s parsability measures for these complex verbs are at odds with what can be inferred from their transitional probabilities ratios and with compositionality scores as well. Hay’s way of assessing decomposability status suggests that *za-rezatj*, with its score of 0.19, is very likely to be parsed (and thus should encode spatial meaning), and *ot-rezatj*, with its score of 1.009, is more likely to be processed holistically (and thus should encode idiosyncratic meaning). A comparison of the two proposed predictive models can help determine whether this inconsistency is a random fluctuation or is indicative of some problems with either measure.

The process of creating the models ran as follows. For each of the 2,566 prefixed verbs in my data, three numerical values were obtained: (1) the logDice compositionality score, (2) the transitional probabilities ratio (log-transformed), and (3) the derivation to base frequency ratio (log-transformed). The data were randomly split into training (80% of observations) and test (20% of observations) sets, and two linear regression models were fit to the training set. Model M regressed compositionality scores on transitional probabilities ratios, whereas Model H regressed compositionality scores on derivation to base frequency ratios. The models’ coefficients can be found in Table 8 (both models were fitted so as to allow for each prefix’s specific baseline, but the coefficients for these factor levels are omitted to avoid clutter).

**Table 8.** Coefficients of the Regression Models.

Model M				Model H			
Coefficient	Estimate	*SE*	*p*	Coefficient	Estimate	*SE*	*p*
Constant	−.23	.33	.42	Constant	−.63	.36	.08
TP ratio	−.46	.02	< 0.001	DB ratio	.18	.01	< 0.001
Prefixes	. . .	. . .	. . .	Prefixes	. . .	. . .	. . .

*Note.* (Model M): *F* = 38.6, *R*^2^ = .24, *p* < .001; (Model H): *F* = 17.7, *R*^2^ = .12, *p* < .001.

**Figure 4.**
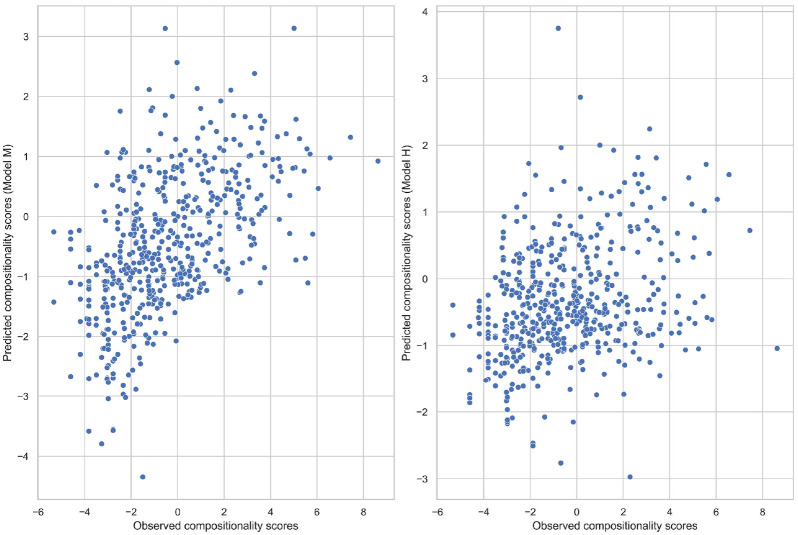
Correlation of the observed and predicted compositionality scores for two models.

The obtained coefficients from both models were used to make separate predictions about the compositionality scores in the test dataset. The predicted and observed compositionality scores were correlated with each other. The resulting plots are presented in Figure 4: the left subplot represents Model M, and the right subplot represents Model H. The correlation coefficients of the observed and predicted compositionality scores were found to be significant in both cases (both *p*-values < .001). However, the strengths of the relationships are not the same: *r* = .52 for Model M and *r* = .30 for Model H. Hence, Model M makes more accurate predictions about the prefixed verbs’ compositionality measures compared with Model H.

The most interesting difference between Models M and H, however, is in the values of the slope coefficients. Model M predicts that with a 1-unit increase in transitional probabilities ratio, the compositionality score will decrease by a factor of −0.46. This is exactly the relationship direction that I expected to find: greater values of the transitional probabilities ratio indicate that for the respective verbs, P (prefix | base) > P (base | prefix), which, in turn, can be taken as a sign that these verbs instantiate constructions of the form **PREFIX** **+** **[_____]_BASE:(X_** _**>)V**_, where the lexical meaning of the base only provides a necessary specification for the general constructional meaning.

Model H, however, predicts that with a 1-unit increase in the derivation to base frequency ratio, the compositionality score will increase by a factor of 0.18. Basically, it implies that the less decomposable a word is, the more compositional its meaning will be. This is counterintuitive. The explanation of this anomaly is, however, very simple. Russian prefixed verbs encoding spatial meanings tend to have a higher token frequency than verbs encoding non-spatial meanings: I found a significant positive correlation between the compositionality scores and frequency values of the derived forms in my data (ρ = 0.64, *p* < .001). Thus, the phenomenon observed with the verbs *ot-rezatj* and *za-rezatj* (Table 7) was not some random fluctuation but rather a manifestation of the fact that derivation to base frequency ratio may be a biased measure.

**Figure 5.**
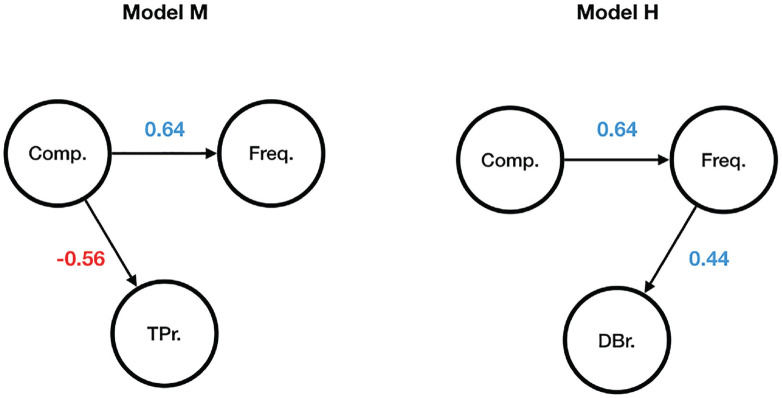
Probabilistic graphical Models M and H.

One can make sense of the ultimate difference between Models M and H by comparing the probabilistic graphical models presented in Figure 5, where *Comp.* stands for the logDice compositionality score, *Freq.* for the token frequency, *TPr.* for the transitional probabilities ratio, and *DBr.* for the derivation to base frequency ratio. Directed edges between the nodes indicate that one node is assumed to exert influence upon another node, whereas the absence thereof means that no direct relation between two nodes is believed to exist. The numbers are the correlation coefficients.

Given the structure of the two probabilistic graphical models in Figure 5, it is easy to see that once the token frequency of a particular complex word is observed, its derivation to base frequency ratio does not provide any information about whether the word’s meaning is compositional or not (the path between the nodes *Comp*. and *DBr*. is blocked). However, once the compositionality value of a particular complex word is known, its token frequency no longer influences its transitional probabilities ratio, that is, one can observe P (prefix | base) ⩽ P (base | prefix) or P (prefix | base) > P (base | prefix) for both high- and low-frequency words.

## 4 Conclusion

Both experimental and corpus data presented in this paper suggest that there exist at least two different constructions for Russian complex verbs: **[_____]_PREFIX_** **+** **BASE** for verbs with spatial meanings and **PREFIX** **+** **[_____]_BASE:(X_** _**>)V**_ for verbs with idiosyncratic meanings.

In study 1, I provided experimental evidence that native speakers, when asked to manipulate complex verbs by changing either their prefix or their base, reveal a significant preference for changing the prefixes of spatial verbs and the bases of non-spatial verbs. In study 2, I showed that the choice of construction could be predicted by taking into account the discrepancy in probabilities of transition from base to affix and from affix to base. If P (prefix | base) ⩽ P (base | prefix), the first construction with an empty slot for the prefix where both elements in combination contribute to the general, additive meaning is likely to be chosen. If, however, P (prefix | base) > P (base | prefix), the second construction with an empty slot for the base becomes more likely. In the latter case, the element of the construction that tells us less about its counterpart activates general constructional meaning, whereas the element that has greater predictive power serves as a filler for the construction’s empty slot. I also showed that the distinction between two constructions does not necessarily pertain to the difference in relative frequencies. Token frequencies of bases and lemmas seem to play hardly any role in either of these constructions. Even low-frequency bases may combine with many different prefixes, and low-frequency lemmas adhere to the same constructional patterns as their high-frequency counterparts.

The distinction between two constructions is easily explainable within the framework of construction morphology (Booij, 2010), where complex words are seen as constructions on the word level. The view that complex words instantiate morphological constructions can be found in Croft (2001) and Goldberg (2006). Some examples of the constructional analysis of complex words include the analysis of English *be*-verbs in Petre and Cuyckens (2008), the analysis of the phrasal verbs of Germanic languages in Booij (2010), and the analysis of Russian prefixed verbs in Monakhov (2021). Nevertheless, overall, the understanding of the constructional aspects of multi-morphemic word structure is still in its early stages.

One non-trivial contribution to the construction morphology framework made by the current study is the idea that some constructions might arise by way of generalization over others, which leads to a shift in the positioning of a fixed element and a slot. These results allow us to draw two important conclusions. First, the distinction between two constructions is not a clear-cut categorical one. Rather, there is an underlying probabilistic continuum, and any particular word can be more or less likely to activate either model of meaning processing. Second, there is compelling evidence that constructions with an empty slot for the base are later developments, compared with their compositional counterparts, as the former come into existence by means of analogy and categorization of the lexical material provided by the latter.

Some limitations of this study must be discussed. First, the potential impact of the examined verbs’ polysemy remains unaccounted for. For example, in Russian, there are two instances of the verb *za-brositj*: (1) “throw something behind some object” and (2) “stop doing something.” The first one is clearly a compositional expression, an instantiation of the **[_____]_PREFIX_** **+** **BASE** construction. It is characterized by a high logDice score and, in the experimental setting described in study 1, would most probably produce suggestions like *na-brositj* “throw on(to) something,” *v-brositj* “throw in(to) something,” *pod-brositj* “throw under something,” and so on. In contrast, the second exemplar is an instantiation of the **PREFIX** **+** **[_____]_BASE:(X_** _**>)V**_ construction with a simple aspectual meaning. It hardly ever collocates with the respective preposition *za* and, when tested experimentally, would most probably evoke variants like *za-konchitj, za-vershitj*, or *za-kljuchitj*, all with the same meaning “bring something to an end.” Unfortunately, with the methodological toolbox of this study, there is no simple way of disambiguating such instances. However, I do not consider this issue to detract from the main conclusions: all Russian prepositional prefixes have developed clear aspectual, meaning-devoid uses, and so any errors in my corpus scores should be distributed across prefixes in an unbiased manner.

Second, it is unclear how the proposed distinction between two constructions can account for Russian prefixed verbs whose prefixes do not have prepositional counterparts. The etymology of these prefixes is not always self-evident, and the patterns of their co-occurrences with prepositions are not always consistent. Some may encode spatial meanings corresponding to the meanings of prepositions that do not coincide with these prefixes in form (*vy-vesti iz doma* “to lead out of the house”). Other prefixes cannot encode spatial meanings at all (*raz-bitj na chasti* “break to pieces”).

Third, the abundance of English and German examples provided in the introduction and elsewhere in this paper should not mislead the reader. Right now, I do not know whether the framework proposed in the study readily extends to German and English data. As already stated, I expect that similar results would be observed in these languages, but this demands further investigation. Thus, the discussion of patterns in languages other than Russian is meant only as a direction for future work.

## Appendix

**Appendix:** Experimental stimuli.

#	Prefix	Verb	Meaning
1	de-	deshifrovatj	decipher
2	dis-	diskvalifitsirovatj	disqualify
3	do-	doezditj	ruin, spoil
4	do-	doplatitj	pay extra
5	do-	dochitatj	finish reading
6	iz-	izgnatj	drive out, expel
7	iz-	izlechitj	heal, cure
8	iz-	izranitj	hurt, wound
9	iz-	ispisatj	cover with writing
10	iz-	issohnutj	become thin, wither
11	na-	nagladitj	iron a lot
12	na-	nakleitj	glue on
13	na-	nalipnutj	stick to
14	na-	nalovitj	catch a lot
15	na-	napetj	hum
16	na-	nasmeshitj	make laugh
17	na-	nauchitj	teach
18	nad-	nadvjazatj	tie, bind above smth
19	nad-	nadpilitj	make an incision
20	nedo-	nedozharitj	undercook
21	niz-	nizvergnutj	overthrow
22	o-	obezhatj	run around
23	o-	okutatj	envelop
24	o-	oprositj	interrogate
25	o-	ochistitj	clear
26	ob-	obvenchatj	marry
27	ob-	obdelitj	deprive
28	ob-	obzharitj	fry
29	ob-	obletetj	fly around
30	ob-	obskakatj	get to windward of smb
31	ob-	objehatj	go around
32	ot-	otblagodaritj	thank
33	ot-	otbrositj	throw away
34	ot-	otgovoritj	talk out of smth
35	ot-	otgremetj	stop rumbling
36	ot-	otdavitj	press hard, crush
37	ot-	otkolotj	break off
38	ot-	otregulirovatj	adjust
39	ot-	otrepetirovatj	rehearse
40	ot-	ottaschitj	drag away
41	pere-	perebuditj	wake up many people
42	pere-	peredelatj	redo
43	pere-	perezhdatj	wait out
44	pere-	perekuritj	smoke for a while
45	pere-	perepilitj	saw through
46	pere-	perepljasatj	outdance
47	pere-	peresypatj	sprinkle with smth
48	pere-	peretrusitj	get the wind up
49	pere-	perehvalitj	overpraise
50	pere-	perehotetj	stop wanting
51	po-	pobezhatj	start running
52	po-	poobedatj	dine
53	po-	poportitj	spoil
54	po-	porabotatj	work for a while
55	po-	posazhatj	plant / put in prison
56	pod-	podbodritj	cheer smb up
57	pod-	podbrositj	toss up
58	pod-	podkopitj	save up
59	pod-	podlizatj	suck up
60	pod-	podmesti	sweep, broom
61	pod-	podpetj	sing along
62	pod-	podplytj	swim near to smth
63	pod-	podsestj	sit next to smth
64	pod-	podslushatj	overhear, eavesdrop
65	pre-	preuvelichitj	exaggerate
66	pred-	predvidetj	foresee
67	pred-	predstavitj	introduce / imagine
68	pri-	pribresti	wander into
69	pri-	prikupitj	buy additionally
70	pri-	prisvistnutj	whistle
71	pri-	pristyditj	put to shame
72	pri-	pritormozitj	slow down
73	pro-	progryztj	gnaw through
74	pro-	produmatj	think through
75	pro-	proehatj	drive through
76	pro-	prozhdatj	wait for a long time
77	pro-	prozvuchatj	resound
78	pro-	propitj	drink away
79	pro-	prospatj	oversleep
80	pro-	proshagatj	walk for some time
81	raz-	razgljadetj	discern
82	raz-	razmorozitj	unfreeze
83	raz-	raskritikovatj	chastise, reprimand
84	raz-	raskroshitj	crumble
85	raz-	rastsvesti	bloom
86	re-	reinterpretirovatj	reinterpret
87	s-	sbritj	shave off
88	s-	sgoretj	burn down
89	s-	skleitj	glue together
90	s-	smjagchitj	soften, attenuate
91	s-	shoditj	visit
92	so-	sosuschestvovatj	live together
93	u-	ugnatj	steal, hijack
94	u-	uzhatj	squeeze
95	u-	ukachatj	become seasick
96	u-	umestitj	fit, squeeze in
97	u-	uplesti	gobble
98	u-	ustavitj	clutter
99	u-	ustyditj	shame
100	u-	uterpetj	endure
101	v-	vkatitj	roll in
102	v-	vpolzti	crawl in
103	voz-	vozvesti	erect
104	voz-	vozlikovatj	rejoice
105	voz-	vozmuzhatj	mature
106	voz-	vozroditj	revive
107	vy-	vybelitj	bleach
108	vy-	vykopatj	dig out
109	vy-	vylovitj	fish out
110	vy-	vylomatj	break out
111	vy-	vysidetj	sit out
112	vz-	vzbesitj	infuriate
113	vz-	vzvolnovatjsja	get excited
114	vz-	vzdorozhatj	rise in price
115	vz-	vzletetj	take off
116	za-	zabrositj	hurl, toss
117	za-	zavoevatj	conquer
118	za-	zagotovitj	prepare, stock
119	za-	zagremetj	start rumbling
120	za-	zadraznitj	tease, bully
121	za-	zaminirovatj	mine
122	za-	zanesti	bring in
123	za-	zanjuhatj	sniff
124	za-	zapudritj	powder
125	za-	zastiratj	wash out

## Notes

1. Some remarks on notation are as follows. (1) Henceforth I will be using the term “prefix” in these formalisms because my hypothesis was tested exclusively on Russian data. With German, it would be more conventional to replace “prefix” with “preverb.” Applying the model to English verb-particle constructions would require changing not only the name of one element (“particle” instead of “prefix”) but also the linear order of the elements: BASE + **[_____]_PARTICLE_** and **[_____]_BASE:(X_** _**>)V**_ + PARTICLE. (2) The subscript (X >)V in the second formalism is used to denote that the prefix accepts a base of category (X >)V—a verb or something that is converted into a verb.

2. The reason I used word2vec model to obtain the examples in Table 6 is a technical one. The Python implementation of the FastText model that I worked with makes it much harder, from a computational perspective, to find the nearest neighbors by vector, so I chose to resort to word2vec to illustrate the main idea. However, I believe FastText to be more reliable for the purpose of large-scale data analysis.
